# Microstructure and Mechanical Properties of Al–Li Alloys with Different Li Contents Prepared by Selective Laser Melting

**DOI:** 10.3390/ma17030657

**Published:** 2024-01-29

**Authors:** Shuobing Shao, Zhuoheng Liang, Peng Yin, Xinyuan Li, Yongzhong Zhang

**Affiliations:** 1 National Engineering & Technology Research Center for Non-Ferrous Metals Composites, GRINM Group Corporation Limited, Beijing 101407, China; 2 GRINM Metal Composites Technology Co., Ltd., Beijing 101407, China; 3 General Research Institute for Nonferrous Metals, Beijing 100088, China

**Keywords:** selective laser melting, Al–Cu–Li–Mg–Ag–Sc–Zr alloys, heterostructure, tensile mechanical properties

## Abstract

Research on the development of new lightweight Al–Li alloys using a selective laser melting process has great potential for industrial applications. This paper reports on the development of novel aluminum–lithium alloys using selective laser melting technology. Al–Cu–Li–Mg–Ag–Sc–Zr pre-alloyed powders with lithium contents of 1 wt.%, 2 wt.% and 3 wt.%, respectively, were prepared by inert gas atomization. After SLM process optimization, the microstructure and mechanical properties of the as-printed specimens were investigated. The densifications of the three newly developed alloys were 99.51%, 98.96% and 92.01%, respectively. They all had good formability, with the lithium loss rate at about 15%. The as-printed alloy with 1% Li content presented good comprehensive properties, with a yield strength of 413 ± 16 MPa, an ultimate tensile strength of 461 ± 12 MPa, and an elongation of 14 ± 1%. The three alloys exhibited a layered molten pool stacking morphology and had a typical heterostructure. The columnar crystals and equiaxed fine grains were alternately arranged, and most of the precipitated phases were enriched at the grain boundaries. The change in Li content mainly affected the precipitation of the Cu-containing phase. When the Li content was 1 wt.%, the following occured: θ phase, T_1_ phase and T_B_ phase. When Li increased to 2 wt.%, T_1_ and T_2_ phases precipitated together. When Li reaches 3 wt.%, δ’ phase precipitated with T_2_ phase. This study provides useful guidance for the future SLM forming of new crack-free and high-strength Al–Li alloys.

## 1. Introduction

The pursuit of lightweight design is an eternal goal in the aerospace field. Due to the advantages of low density, good corrosion resistance, high strength and excellent toughness, the application of Al–Li alloys in the aerospace field is gradually replacing the traditional aluminum alloy and playing an increasingly critical role [1,2,3,4,5,6]. Selective laser melting (SLM) has the characteristics of high forming precision and is not limited by the shape of parts [7,8,9,10,11]. It is especially suitable for the rapid forming of complex precision parts and provides an effective way to facilitate the lightweight design of structures. Therefore, the rapid preparation of Al–Li alloy integral structural parts by SLM technology at a low cost meets the development needs of the aerospace field and has high research value [12,13,14].

The most widely studied aluminum alloys in SLM are Al–Si cast aluminum alloys, of which AlSi10Mg has received the most attention [15,16]. These Al–Si casting aluminum alloys have good SLM process properties due to their narrow solidification range and low shrinkage [16,17]. In the case of commonly used deformed aluminum alloys such as 2xxx, 5xxx, 6xxx and 7xxx series alloys, attempts to use them for SLM usually result in thermal cracking [18,19,20,21]. Conventional alloys designed for casting and deformation processes are unable to take full advantage of the potential benefits of SLM’s high solidification rates. As a result, the focus of research on SLM aluminum alloys has gradually shifted from cast and deformed aluminum alloys to the development of new aluminum alloy systems that can be adapted to the unique thermomechanical conditions of the SLM process. The first commercial aluminum alloy specifically designed for SLM may be the Scalmalloy alloy (Al-4.60Mg-0.66Sc-0.42Zr-0.49Mn, wt.%) [22]. SLM-formed Scalmalloy alloys are characterized by fine grain size and high tensile properties [23,24,25,26,27]. The results of SLM processing of commercial alloys point to the importance of grain refinement in solving the cracking problem. It is noteworthy that the content of inoculation elements such as Sc and Zr is much higher than that of conventional cast and deformed aluminum alloys [28,29,30,31].

The main challenge in developing alloys for SLM is the preparation of pre-alloyed powders with different compositions. The first approach is to mix existing deformed aluminum alloy powders with inoculation powders, and the composition of the mixed powders can be easily adjusted by controlling the amount of inoculation powder [32,33]. However, it may lead to uneven melting of the mixed powder. The second method is to use the ingot to prepare the pre-alloyed powder. The advantage is that the modified elements or in situ enhanced particles can be uniformly distributed in the powder [34,35]. Therefore, the second method is chosen for this topic.

There are fewer research reports on the SLM of Al–Li alloys [36,37]. The Al–Li alloy has a large solidification range and a high thermal expansion coefficient [38,39]. With the addition of Li to the Al–Li alloy, the density of the alloy will first decrease, and then the eutectic grains with low melting point are easily precipitated in the intergranular section during the solidification process [40], resulting in a high hot cracking sensitivity of the alloy. Under the action of a high-energy laser beam, the hot cracking tendency further increases [41,42,43,44]. The difficulty in forming Al–Li alloys by SLM processing can be solved by adding Sc and Zr; this is mainly thanks to the minimal lattice mismatch between the primary Al_3_(Sc, Zr) precipitates and the aluminum alloy matrix, with the resulting low interfacial energy ensuring an effective heterogeneous nucleation point for grain crystallization [45]. These elements form a complex L1_2_-structure-phase Al_3_(Sc, Zr) [46]. Such dispersoids exhibit core-shell structure with Sc-enriched core and Zr-enriched shell [47,48,49,50]. The low-diffusion Zr atoms improve the thermal stability, and the high-diffusion Sc atoms accelerate the nucleation of the dispersed phase [51]. The addition of Sc/Zr elements facilitates the formation of fine equiaxed grains, which can effectively adapt to the strains generated during the SLM process and avoid solidification cracks. The primary Al_3_(Sc, Zr) generated at high cooling speeds can significantly increase the proportion of equiaxial grains and refine the grain. It has been shown that thermal cracks can be eliminated with suitable added amounts of Sc and Zr, and their grain refinement effect can also make the material both strong and tough [52,53].

In this study, three Al–Cu–Li–Mg–Ag–Sc–Zr alloys were designed based on the commercially available 2195 Al–Li alloy. The lithium contents were set at 1%, 2% and 3%, respectively, to reduce the density of the alloys, and the Sc and Zr elements were added to avoid cracks. The focus is on the forming quality, microstructure and mechanical properties of the three SLM-formed alloys.

## 2. Materials and Methods

### 2.1. Powder Preparation

**Figure 1:**
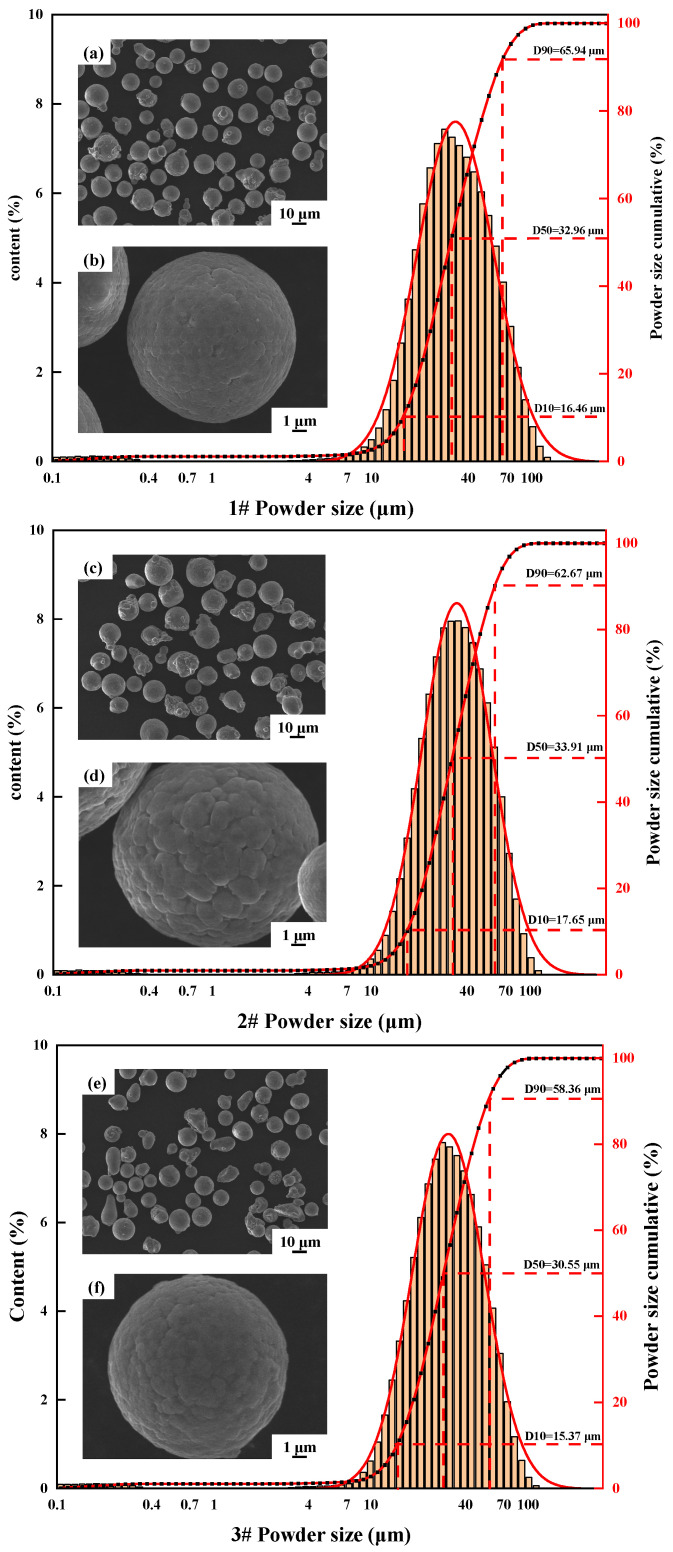
Powder morphology and particle size distribution: (**a**,**b**) 1# alloy powder morphology; (**c**,**d**) 2# alloy powder morphology; (**e**,**f**) 3# alloy powder morphology.

In this experiment, the raw material Al–Cu–Li–Mg–Ag–Sc–Zr powders were prepared by inert gas atomization, and the powder size distribution and morphology of the three alloys are shown in Figure 1. The design composition is shown in Table 1. Powder size is 15–70 µm, and the alloy powder is spherical with few satellites. The alloying elements were determined by gas-pulsed infrared method (GB/T 20975.25-2020 [54]) and inductively coupled plasma atomic emission spectrometry (ICP-AES, Burbach, North Rhine-Westphalia, Germany). For the convenience of the subsequent description, the SLM-formed specimens are named 1#, 2# and 3# alloys according to the level of Li content.

### 2.2. Forming Process

In this experiment, the EP-M250 metal 3D printing system (Beijing e Plus 3D Technology Co., Ltd., Beijing, China) was used, with a maximum laser power of 500 W and a spot diameter of 70 µm. Argon was used as the protective gas, and the oxygen content in the cavity of the 3D printing system was strictly controlled to be less than 100 ppm. The SLM process is accompanied by the interactions between the powder and the laser, and several process parameters affect the quality of the formed alloy, such as volumetric energy density (E), scanning speed (V), layer thickness (D), hatch spacing (H), laser power (P) and preheating temperature of the deposited substrate. Different process parameters also lead to large differences in the forming quality, surface morphology, microstructure, and mechanical properties of the alloys, so calculating the optimal process parameters is the primary objective. Some of the above-mentioned process parameters have the following mathematical relationships [55]:

(1) E=P/(V×H×D)

The preheating temperature of the deposition substrate was set at 140 degrees Celsius, and a parallel scanning strategy was used with 67° rotation per layer in the scanning direction, and a horizontal sample with dimensions of 65 mm × 11 mm × 11 mm was printed for subsequent experiments. The SLM process parameters were set as follows: P = 150~270 W; E = 80~240 J/mm; H = 0.1 mm; and D = 0.03 mm. Following extensive testing, the process parameters with the highest forming density were selected. The surface quality of the specimens under these processes was good, with no cracks. The chemical compositions of the powder and formed samples are shown in Table 2. During the forming process, the loss rate of Li was about 15%. The high temperature generated under the action of the laser led to the evaporation of Li, Mg and other elements with low melting points as well as some Ag, Sc and Zr in the form of soot, which led to a decrease in element content in the formed sample. The theoretical densities of 1#, 2# and 3# alloys were calculated to be 2.64 g/cm^3^, 2.51 g/cm^3^ and 2.41 g/cm^3^, respectively.

### 2.3. Experimental Procedures

Vickers microhardness tests were carried out using an HXD-1000TM (Shanghai Optical Instrument Factory, Shanghai, China) microhardness tester at a pressure of 10 N. In order to improve accuracy, each sample was measured five times. When the density of the formed sample was measured, the sample surface was first polished with sandpaper and then polished with polishing cloth combined with polishing paste. The direct reading solid density meter (SJ-300G, Shanghai Shuju Instrument Technology Co., Ltd., Shanghai, China) was used. Its working principle was the Archimedean drainage principle. The densification of the prepared sample was calculated by calculating the ratio of the measured value to the theoretical density calculated according to the formula. Using X-ray-computed Tomography (CT) (V |tome| × s 240/180, GE Sensing & Inspection Technologies GmbH, Frankfurt, Germany), the defects in the entire sample volume could be highlighted and the density measured by the drainage method could also be verified.

**Figure 2:**
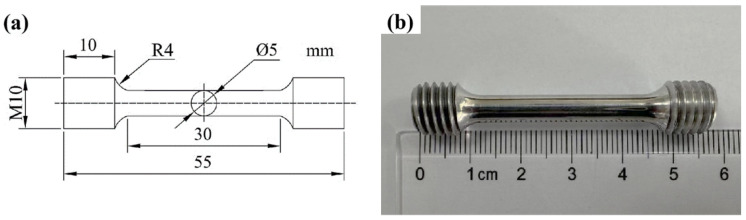
(**a**) Schematic diagram of a tensile bar specimen; (**b**) real sample.

The porosity defects and melt pool morphology inside the formed specimens were observed by an Axiovert 200 MAT optical microscope (OM) after corrosion with Kaller’s reagent for 10~15 s. The phase analysis was carried out using an X’Pert Pro MPD analyzer from Panalytical, Almelo, The Netherlands, with a step size of 0.033° per scan. The microstructure and morphology of the SLM-formed parts were observed and analyzed by a JSM-7900F field emission scanning electron microscope (SEM), and the molten pool morphology, grain size and second phase of the alloy were analyzed by an energy spectrum instrument, electron channel diffraction (ECC), and backscattering diffraction, which were equipped with the equipment. To observe and analyze the grains and the second phase on the micron and even nanometer scale, an FEI G2 60-300 and an FEI Tecnai F20 Transmission Electron Microscope (TEM) and the accompanying energy dispersive spectrometers (EDS) were used. Room temperature tensile tests were performed on an Instron-3382 electronic universal material testing machine, three samples were selected from each group, and the test procedure was carried out according to the test standard GB/T228.1-2010 [56]. The shape and dimensions of the tensile specimens are shown in Figure 2.

## 3. Results and Discussion

### 3.1. Forming Process Optimization

**Figure 3:**
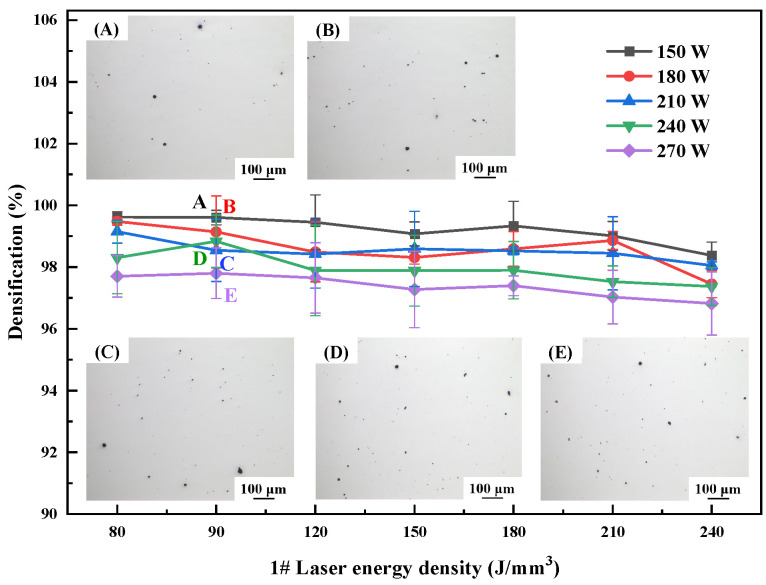
Densification of as-printed 1# alloy at different process parameters.

**Figure 4:**
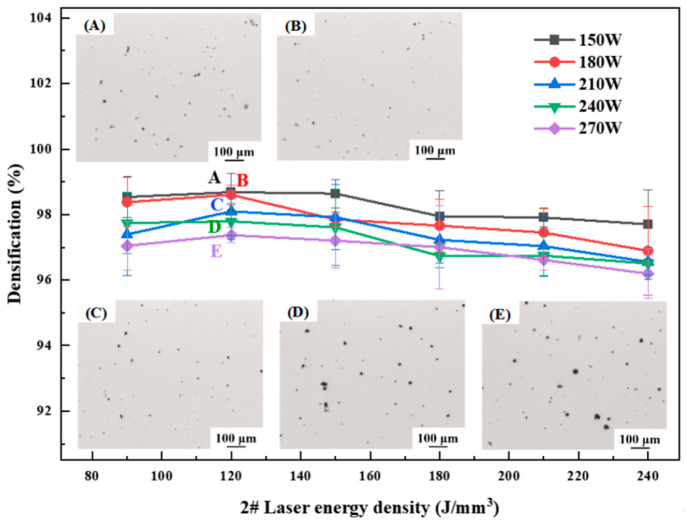
Densification of as-printed 2# alloy at different process parameters.

**Figure 5:**
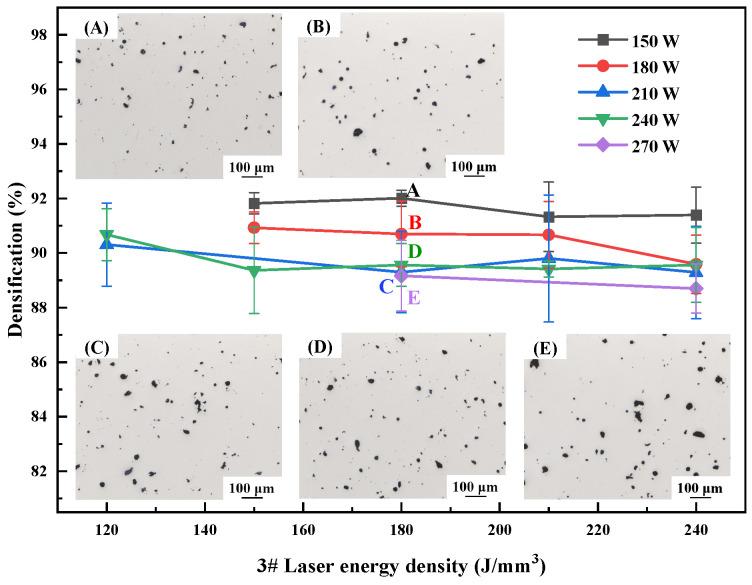
Densification of as-printed 3# alloy at different process parameters.

The optimization of the SLM forming process for different materials is an essential step toward the fabrication of components with good performance. The laser power varies between 150 W, 180 W, 210 W, 240 W, and 270 W, and the energy density varies between 80 J/mm^3^, 90 J/mm^3^, 120 J/mm^3^, 150 J/mm^3^, 180 J/mm^3^, 210 J/mm^3^, and 240 J/mm^3^; for a wide range of process optimizations, a scanning spacing of 0.1 mm and a layer thickness of 0.03 mm were used. Specimen densification is an important indicator of the quality of forming. Figure 3, Figure 4 and Figure 5 show the relationship between energy density and sample densification at the same laser power. The densification of the alloys formed by SLM decreased as the Li content increased; the highest densifications of 99.58%, 99.12% and 93.03% can be obtained, respectively, under the optimal process. The optimized SLM forming processes for these alloys were as follows: 1# alloy (P = 150 W, V = 556 mm/s, E = 90 J/mm^3^), 2# alloy (P = 150 W, V = 417 mm/s, E = 120 J/mm^3^) and 3# alloy (P = 150 W, V = 278 mm/s, E = 180 J/mm^3^). The specimens’ surface quality was good with no cracks. As the laser power or energy density increases, the overall trend of densification decreases, and the SLM forming process is sensitive to the Li content, which leads to a narrow forming process window. High energy density corresponds to low scanning speed, slow cooling speed and long residence time at high temperatures inside the molten pool, which aggravates the volatilization of the low-melting-point elements Li and Mg, thus forming round pores and decreasing the densification of the formed specimens. High laser power will lead to high temperature of the melt pool, and the impact of the laser beam on the melt pool becomes strong, resulting in a large number of melt pool splash droplets. These splash droplets will lead to spherical bumps on the surface, slagging and other phenomena.

**Figure 6:**
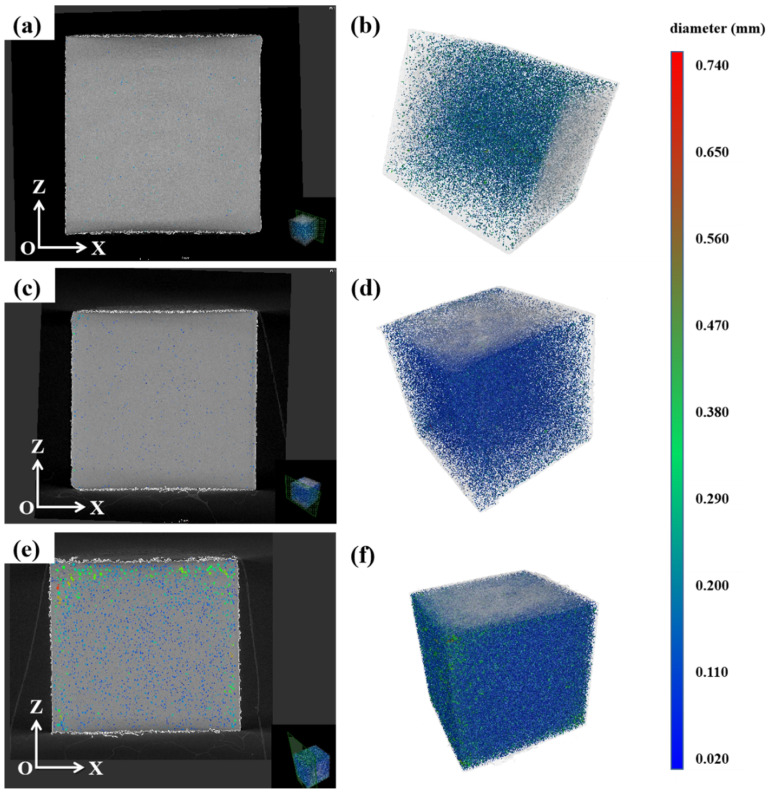
The microscopic defect distribution of XOZ cross section and the whole sample of as-printed alloy: (**a**,**b**) 1#; (**c**,**d**) 2#; (**e**,**f**) 3#.

The highest densification measured by the drainage method was confirmed by industrial CT, and the results are presented in Figure 6. The results show that the densifications of as-printed alloy 1#, 2# and 3# were 99.51%, 98.96% and 92.01%, respectively. The final density was determined by industrial CT. Most of the pores in the sample are spherical, which accords with the characteristics of volatile pores, and a few irregular pores are presumed to be some incomplete melting powder gaps. The rounder the pore shape, the more uniform the stress distribution, the higher the Young’s modulus and the higher the ultimate tensile strength of the material.

### 3.2. Microstructure and Phase Analysis

#### 3.2.1. Microstructure

**Figure 7:**
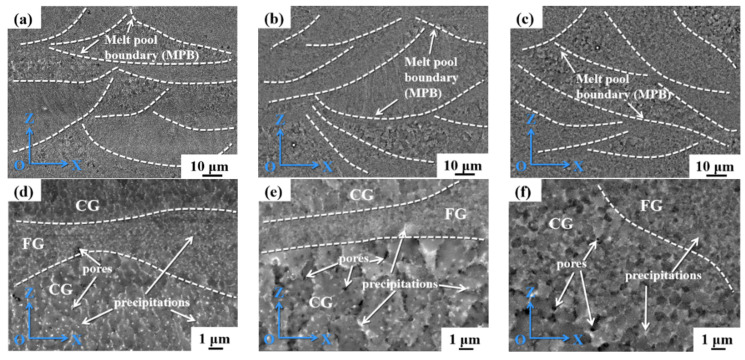
SEM morphology of the as-printed Al–Li alloy: (**a**,**d**) 1#; (**b**,**e**) 2#; (**c**,**f**) 3#.

Figure 7 shows the backscattered electron (BSE) SEM photographs of the samples. The white dashed lines are labeled as melt pool boundaries (MPBs). The SLM solidification process is found to depend on the cooling rate, where a fast-cooling rate leads to coarse columnar grains with a <100> crystal orientation (perpendicular to the MPBs). Columnar epitaxial growth nucleating on the dendritic substrate occurs throughout the as-printed samples. It is worth noting that the large STR also enhances the crack sensitivity during SLM. A large number of white precipitates are visible near grain boundaries (GBs), MPBs, and intracrystalline sections. Due to the high cooling rate of 10^5^–10^7^/s in SLM, the precipitated phases are dispersed.

**Figure 8:**
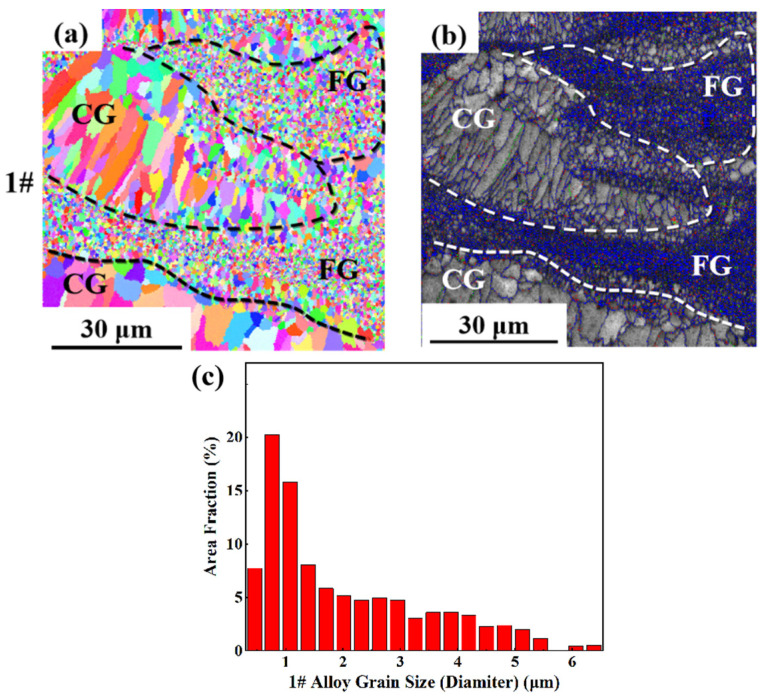
EBSD analysis result of as-printed 1#: (**a**) grain remodeling; (**b**) grain boundary remodeling; (**c**) grain size statistics.

**Figure 9:**
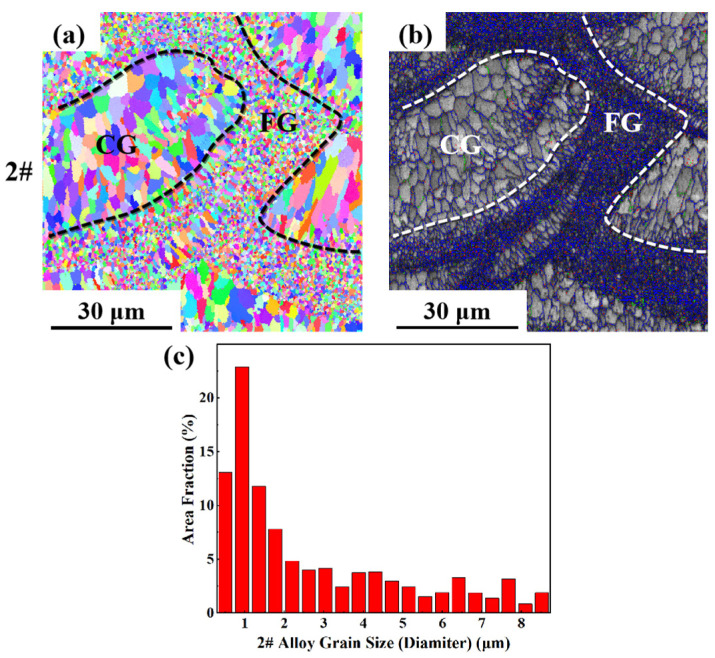
EBSD analysis result of as-printed 2#: (**a**) grain remodeling; (**b**) grain boundary remodeling; (**c**) grain size statistics.

**Figure 10:**
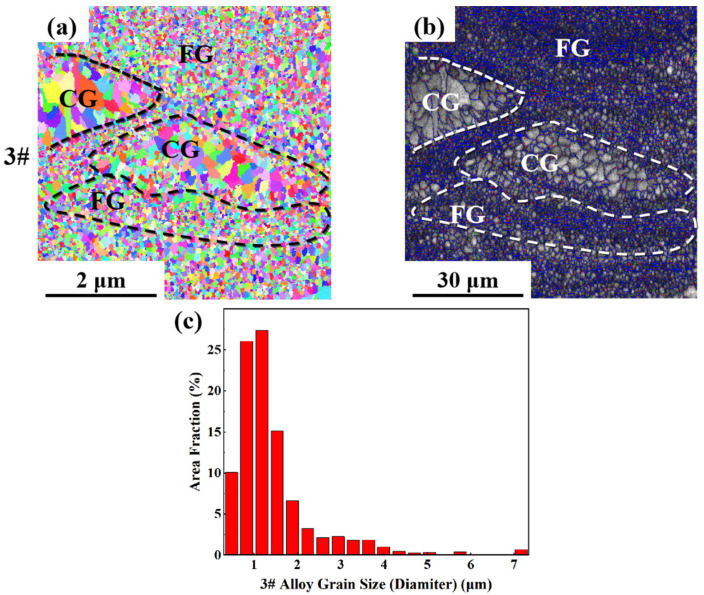
EBSD analysis result of as-printed 3#: (**a**) grain remodeling; (**b**) grain boundary remodeling; (**c**) grain size statistics.

As can be seen from the EBSD morphology in Figure 8, Figure 9 and Figure 10, the as-printed 1#, 2# and 3# alloys have typical heterogeneous structures, i.e., alternating columnar grains (CG) and fine equiaxed grains (FG). The SLM specimens show a bimodal grain structure consisting of fine equiaxial grains and fine columnar grains. Similar microstructures were observed in other Sc- and Zr-modified aluminum alloys prepared by SLM [57,58,59]. Controlled by the forming process parameters and the characteristics of the temperature field inside the molten pool, equiaxed grains are mainly distributed at the boundary of the molten pool, and columnar grains are grown from the boundary of the molten pool to the inside. The top of the molten pool is often due to columnar or equiaxed grain transformation (CET) to form a certain thickness at the equiaxed grain zone. Because the remelting depth of the latter layer is greater than the thickness of the equiaxed grain zone, the equiaxed grain zone will be completely remelted during the deposition process of the next layer, thus forming a columnar grain structure epitaxially grown from the bottom to the top of the structure. With the increase in Li content, the proportion of the grain size distribution of the as-printed alloy in the range of 0~2 μm gradually increases. At the same laser power, as the energy density increases, the scanning speed slows down, which promotes the grain growth. However, in this experiment, with the same laser power, as the energy density increases, the grain size becomes finer with the increase in Li content, which indicates that the increase in Li content plays an absolutely positive role in grain refinement. The reason is that after the Li content increases, many fine Li-containing phases precipitate, providing nucleation sites and promoting grain refinement.

#### 3.2.2. XRD Phase Analysis

**Figure 11:**
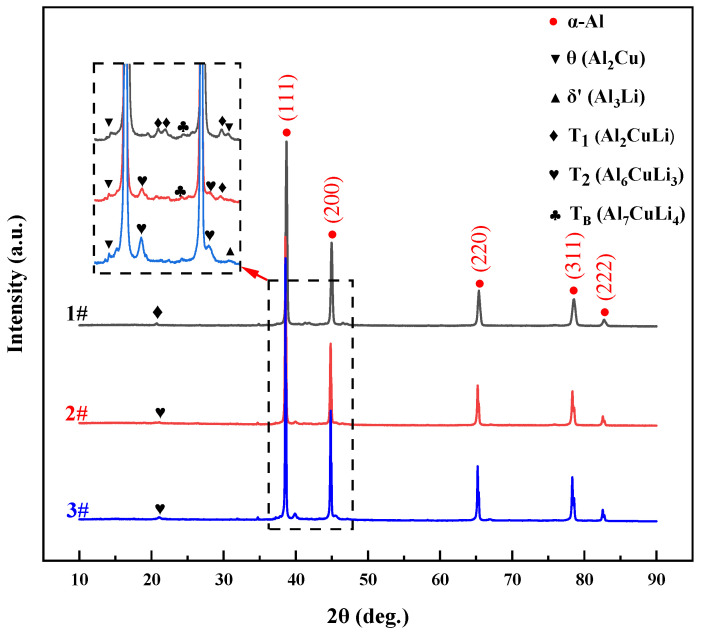
XRD patterns of as-printed Al–Li alloy specimens.

Figure 11 shows the X-ray diffraction (XRD) patterns of the three as-printed alloys. The X-ray diffraction analysis demonstrated the existence of many phases inside the aluminum (α-Al) matrix, including θ (Al_2_Cu), T_1_ (Al_2_CuLi), T_2_ (Al_6_CuLi_3_) and T_B_ (Al_7_CuLi_4_).

All three alloys’ X-ray diffraction spectra displayed distinct and notable α-Al peaks. Diffraction peaks corresponding to Al_3_(Sc, Zr) were not illustrated in the SLM-formed Al–Li alloy. During SLM forming, the solid solubility of Sc and Zr increases markedly. This result is further influenced by the alloys’ low concentrations of Sc and Zr as well as the X-ray diffraction testing’s limited sensitivity.

The variability in Li content is the primary factor that significantly affects the formation of Cu-containing phases. The precipitation of the θ (Al_2_Cu) phase, T_1_ (Al_2_CuLi) phase and T_B_ (Al_7_CuLi_4_) phase is favored from both the α-Al phase and the liquid phase when the Li content is 1.0 wt.%. Upon further increasing the lithium content to about 2.0 wt.%, the T_1_ phase is precipitated in conjunction with the T_2_ (Al_6_CuLi_3_) phase. When the Li content reaches 3 wt.%, the T_1_ phase no longer precipitates, and the δ’ (Al_3_Li) phase precipitates with the T_2_ phase. The precipitation process can be briefly outlined as follows: θ → T_B_ → T_1_ → T_2_ → T_2_ + δ’.

### 3.3. TEM Morphology and Analysis

**Figure 12:**
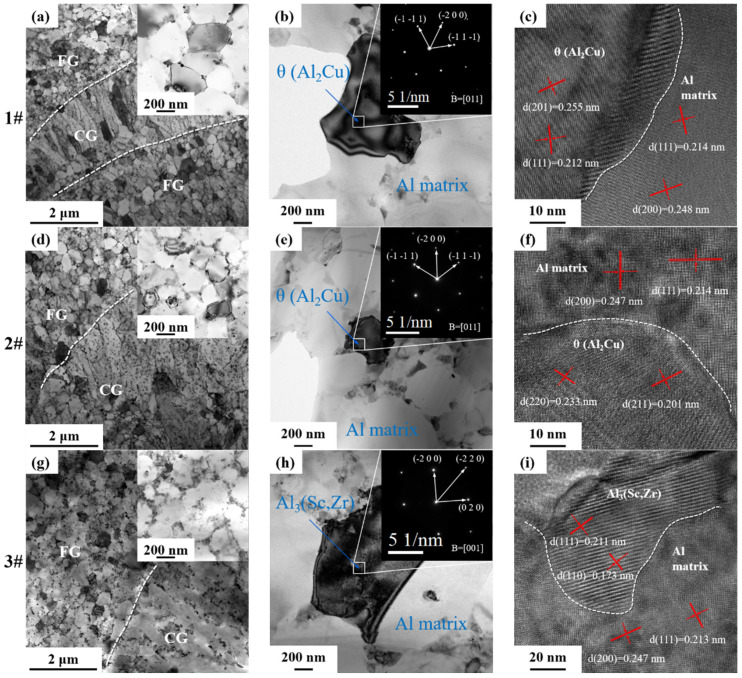
The morphological characteristics of equiaxed and columnar crystals of as-printed under STEM: bright field (**a**,**d**,**g**); SAED pattern index and relative orientation relationship (ORs) (**b**,**e**,**h**); HRSTEM image of the interface between the precipitated phase and the Al matrix (**c**,**f**,**i**).

Figure 12 shows the microstructure of the interface between equiaxed grains and columnar grains in the molten pool of the as-printed alloy, TEM photos and diffraction analysis and TEM high-resolution photos.

From the bright-field image of Figure 12a,d,g, it can be seen that the size of the equiaxial grains is in the range of 0.5~1 μm, and the length of the columnar grains is in the range of about 2~5 μm. There are continuous strips of precipitates at the junction of the grains; many nanoscale particles are found within the grains of the columnar grains and equiaxial grains. In Figure 12b,e,h, the SAED patterns of θ (Al_2_Cu) phase and primary Al_3_(Sc, Zr) phase of the three Al–Li alloys are indexed to show the relative orientation relationships (ORs). From the HRSTEM image of Figure 12c,f,i, the interface between the precipitated phase and the Al matrix can be seen. There is a certain dislocation between the precipitated phase and the atoms’ neighbor to the aluminum matrix. The dislocation cuts through the particles; the particles produce a new surface area, which increases the total interface energy; and the atoms in the particles are staggered, which brings difficulties for the movement of dislocations and plays a strengthening effect.

**Figure 13:**
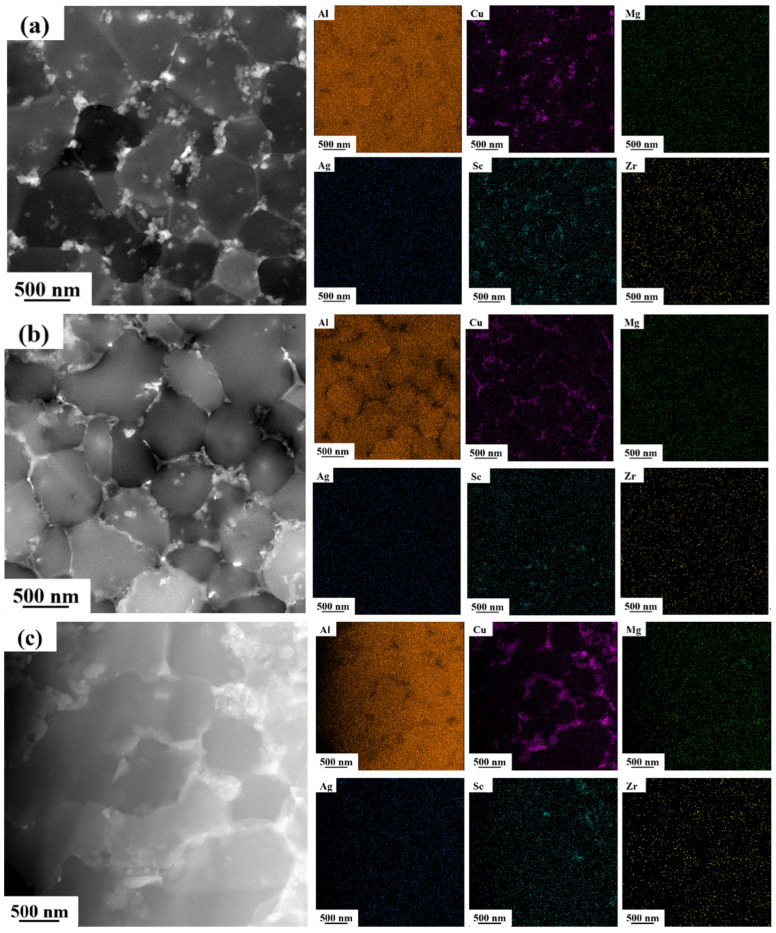
STEM HAADF images of as-printed samples corresponding to EDX mapping of the solute elements (Al, Cu, Mg, Ag, Sc, Zr): (**a**) 1#; (**b**) 2#; (**c**) 3#.

Figure 13a–c shows the distribution of Al, Cu, Mg, Ag, Sc and Zr elements in 1# 2# and 3# alloys determined by high-angle annular dark field (HAADF) images. It is well known that the elemental distribution of lithium cannot be directly detected by EDS or WDS. The distribution of Li-containing precipitates can be studied and speculated upon according to the distribution of other elements by EDS surface scanning. Most of the Cu and Sc content is precipitated at the grain boundaries and cover part of the grain boundaries. The distribution of the Mg, Ag and Zr elements is more uniform. The cooling rate at the bottom of the molten pool is lower than that inside the molten pool. The remelting at the bottom of the molten pool and the low overheating and slightly lower cooling rate led to the precipitation of primary Al_3_(Sc, Zr) at the bottom, and these primary Al_3_(Sc, Zr) can be used as nucleation centers to form equiaxed grains [37,60,61,62]. Columnar grains grow from the outer edges of the equiaxed grains and may introduce some incipient Al_3_(Sc, Zr) [35,63].

The intensity of the images in HAADF is positively correlated with the atomic mass, and the analysis shows the presence of precipitates with different contrasts (white, gray and black) in a single medium. Combined with the EDS results, the white precipitates are rich in Cu. These white precipitates are elliptical or small flakes, mostly located at grain boundaries, and are presumed to be T_1_ or T_B_ phases containing Li elements.

### 3.4. Mechanical Propertie

**Figure 14:**
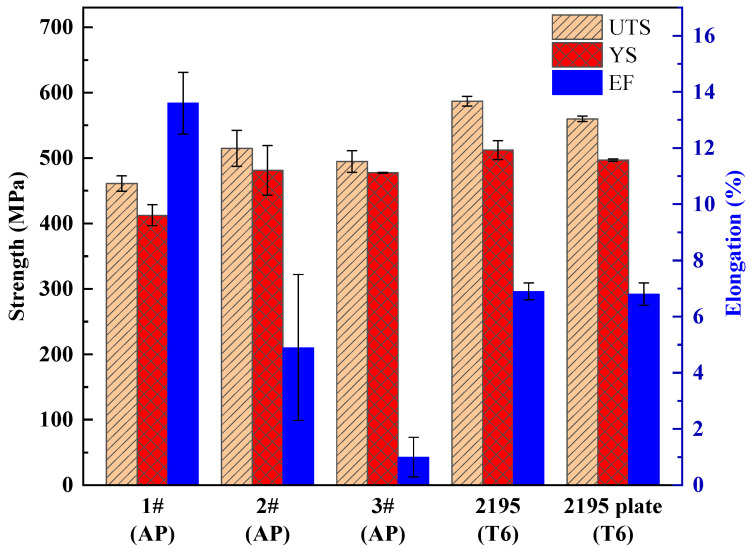
The tensile properties of as-printed 1#, 2#, 3# alloys and SLM-formed 2195 alloy and 2195 plate after T6 heat treatment were compared with the histogram.

The microhardness of the three as-printed alloys was measured and compared, and five points were selected for each sample; the hardness values of 1#, 2# and 3# alloys are 135 ± 3 HV, 160 ± 3 HV and 148 ± 4 HV, respectively. The mechanical properties of the as-printed state of the three compositions are shown in Table 3 and Figure 14. The tensile properties of the as-printed state of the three compositions are close to those of the SLM-formed 2195 alloy and commercial 2195 plate-T6 [41,64], which require an aging time of up to 180 h. The reasons for the higher strength of the alloy are as follows. First, the grains are relatively small. Fine grains at room temperature have higher strength, hardness, plasticity and toughness. This is because the fine grains are plastically deformed by external forces and can be dispersed in more grains. The plastic deformation is more uniform and the stress concentration is smaller. In addition, the finer the grain size, the larger the grain boundary area, the more tortuous the grain boundary and the more unfavorable the crack propagation. Second, the SLM process can make Al–Li alloys make good use of Sc and Zr when the solid solubility exceeds the equilibrium, and it can enhance grain boundary strengthening through grain refinement. The good mechanical properties are attributed to the solid solution strengthening of Sc and Zr with a small amount of Li in the Al matrix. As a grain refiner, Al_3_(Sc, Zr) particles provide nucleation particles for the nucleation of the matrix and control the grain size. Many grain boundaries hinder the initiation of solidification cracks and enhance the plasticity and toughness. With the increase in Li content, the hardness, tensile strength and yield strength of 1#, 2# and 3# alloys increase first and then decrease, and the elongation and reduction of area decrease. The reason is that the main strengthening phase of the Al–Li alloy is T_1_ phase. When the Li content is increased to 2 wt.%, the reason for the improvement in performance is that the T_1_ of the as-printed alloy is found to increase by XRD. At 3 wt.%, the performance degradation is mainly due to the transformation of the main strengthening phase T_1_ in the alloy into T_2_ and δ’. Secondly, the increase in porosity in the alloy will also reduce the performance.

## 4. Conclusions

This paper studies the SLM formation of Al–Cu–Li–Mg–Ag–Sc–Zr alloys with different Li contents. The differences in porosity, microstructure and mechanical properties of the as-printed specimens of the three alloys are compared. The main conclusions are as follows:

1. The alloys with different Li contents successfully prepared by SLM technology are crack-free, with good surface quality and high densifications of 99.51%, 98.96% and 92.01%, respectively.
2. With the increase in laser power and energy density, the density decreases gradually. At high energy density, the cooling rate of the molten pool is slow and the internal high temperature residence time is long, which aggravates the volatilization of the low-melting-point elements Li and Mg, thus forming circular pores, resulting in reduced density.
3. When the Li content is 1.0 wt.%, θ phase, T_1_ phase and T_B_ phase occur; after 2.0 wt.%, the T_1_ and T_2_ phases are precipitated together; and at 3.0 wt.%, the δ’ phase precipitates together with the T_2_ phase.
4. With the increase in Li content, the hardness, tensile strength and yield strength of 1#, 2# and 3# alloys increase first and then decrease, and the elongation and reduction in area decrease. The reason is that the change in Li content affects the T_1_ phase and the pore defects in the as-printed alloy.

The Li content in the existing Al–Li alloys is less than 2.5 wt.%, but the maximum solubility of Li in the α-Al phase is 4.2 wt.%. The slow cooling rate during casting will form a brittle-phase δ phase, which is unfavorable to mechanical properties and corrosion resistance. In contrast, the cooling rate of SLM (10^3^ K/s~10^6^ K/s) is extremely fast, and Li can be uniformly distributed in the α-Al phase, avoiding the formation of the δ phase, so that Al–Li alloys with high Li content can be formed by the SLM process. The crack control was realized by optimizing the process parameters and refining the grains by microalloying, adding Sc and Zr to form Al_3_(Sc, Zr) particles in the alloy as a non-uniform nucleating agent. With their potential for use in various applications, the rapid manufacturing and development of new Al–Li alloy structural parts through the SLM process could foster future development in the aerospace industry.

## Figures and Tables

**Table 1:** The design composition of Al–Cu–Li–Mg–Ag–Sc–Zr powder.

Elements/wt.%	Cu	Li	Mg	Ag	Sc	Zr	Al
2195	3.7~4.3	0.8~1.2	0.25~0.8	0.25~0.6	/	0.08~0.16	Bal.
1#	4 ± 0.2	1 ± 0.2	0.6 ± 0.2	0.5 ± 0.1	0.8 ± 0.1	0.4 ± 0.1	Bal.
2#	4 ± 0.2	2 ± 0.2	0.6 ± 0.2	0.5 ± 0.1	0.8 ± 0.1	0.4 ± 0.1	Bal.
3#	4 ± 0.2	3 ± 0.2	0.6 ± 0.2	0.5 ± 0.1	0.8 ± 0.1	0.4 ± 0.1	Bal.

**Table 2:** Composition of raw powder and as-printed samples.

Elements/wt.%		Cu	Li	Mg	Ag	Sc	Zr	Al
Raw powder	1#	3.78 ± 0.2	1.24 ± 0.3	0.59 ± 0.2	0.40 ± 0.1	0.80 ± 0.1	0.42 ± 0.1	Bal.
	2#	3.92 ± 0.2	2.67 ± 0.3	0.67 ± 0.2	0.53 ± 0.1	0.76 ± 0.1	0.42 ± 0.1	Bal.
	3#	3.90 ± 0.2	3.71 ± 0.3	0.66 ± 0.2	0.58 ± 0.1	0.76 ± 0.1	0.52 ± 0.1	Bal.
As-printed sample	1#	4.08 ± 0.2	1.16 ± 0.3	0.50 ± 0.2	0.38 ± 0.1	0.76 ± 0.1	0.41 ± 0.1	Bal.
	2#	4.18 ± 0.2	2.26 ± 0.3	0.47 ± 0.2	0.42 ± 0.1	0.74 ± 0.1	0.42 ± 0.1	Bal.
	3#	4.07 ± 0.2	3.24 ± 0.3	0.50 ± 0.2	0.48 ± 0.1	0.72 ± 0.1	0.52 ± 0.1	Bal.

**Table 3:** The tensile properties of as-printed 1#, 2#, 3# alloys were compared with as-printed 2195 alloy and 2195 plate after T6 heat treatment.

Samples	UTS (MPa)	YS (MPa)	Elongation (%)	Modulus (GPa)
1# AP	461 ± 12	413 ± 16	14 ± 1	73 ± 2
2# AP	515 ± 28	481 ± 38	5 ± 3	76 ± 3
3# AP	495 ± 17	478 ± 1	1 ± 1	74 ± 1
2195 T6 [41]	587 ± 8	512 ± 14	7 ± 1	/
2195 plate-T6 [64]	560 ± 4	497 ± 2	7 ± 1	/

## Data Availability

Date are contained within the article.

## References

[1] Li S.S., Yue X., Li Q.Y., Peng H.L., Dong B.X., Liu T.S., Yang H.Y., Fan J., Shu S.L., Qiu F. (2023). Development and applications of aluminum alloys for aerospace industry. J. Mater. Res. Technol..

[2] Kablov E.N., Antipov V.V., Oglodkova J.S., Oglodkov M.S. (2021). Development and Application Prospects of Aluminum–Lithium Alloys in Aircraft and Space Technology. Metallurgist.

[3] Rioja R.J., Liu J. (2012). The Evolution of Al-Li Base Products for Aerospace and Space Applications. Metall. Mater. Trans. A.

[4] Ahmed B., Wu S.J. (2013). Aluminum Lithium Alloys (Al-Li-Cu-X)-New Generation Material for Aerospace Applications. Appl. Mech. Mater..

[5] Wanhill R.J.H. (2014). Aerospace Applications of Aluminum–Lithium Alloys, Aluminum-Lithium Alloys.

[6] Abd El-Aty A., Xu Y., Guo X., Zhang S.H., Ma Y., Chen D. (2018). Strengthening mechanisms, deformation behavior, and anisotropic mechanical properties of Al-Li alloys: A review. J. Adv. Res..

[7] Yap C.Y., Chua C.K., Dong Z.L., Liu Z.H., Zhang D.Q., Loh L.E., Sing S.L. (2015). Review of selective laser melting: Materials and applications. Appl. Phys. Rev..

[8] Seabra M., Azevedo J., Araújo A., Reis L., Pinto E., Alves N., Santos R., Pedro Mortágua J. (2016). Selective laser melting (SLM) and topology optimization for lighter aerospace componentes. Procedia Struct. Integr..

[9] Frazier W.E. (2014). Metal Additive Manufacturing: A Review. J. Mater. Eng. Perform..

[10] Sames W.J., List F.A., Pannala S., Dehoff R.R., Babu S.S. (2016). The metallurgy and processing science of metal additive manufacturing. Int. Mater. Rev..

[11] Lewandowski J.J., Seifi M. (2016). Metal Additive Manufacturing: A Review of Mechanical Properties. Annu. Rev. Mater. Res..

[12] Xu R., Li R., Yuan T., Niu P., Wang M., Lin Z. (2020). Microstructure, metallurgical defects and hardness of Al–Cu–Mg–Li–Zr alloy additively manufactured by selective laser melting. J. Alloys Compd..

[13] Raffeis I., Adjei-Kyeremeh F., Vroomen U., Richter S., Buhrig-Polaczek A. (2020). Characterising the Microstructure of an Additively Built Al-Cu-Li Alloy. Materials.

[14] Sun Z., Wang H., Tian X., He B. (2023). Developing a novel lightweight Al–Mg–Li alloy for laser powder bed fusion additive manufacturing: Parameter optimization, microstructure evolution, and mechanical performance. Mater. Sci. Eng. A.

[15] Trevisan F., Calignano F., Lorusso M., Pakkanen J., Aversa A., Ambrosio E.P., Lombardi M., Fino P., Manfredi D. (2017). On the Selective Laser Melting (SLM) of the AlSi10Mg Alloy: Process, Microstructure, and Mechanical Properties. Materials.

[16] Kempen K., Thijs L., Van Humbeeck J., Kruth J.P. (2012). Mechanical Properties of AlSi10Mg Produced by Selective Laser Melting. Phys. Procedia.

[17] Brandl E., Heckenberger U., Holzinger V., Buchbinder D. (2012). Additive manufactured AlSi10Mg samples using Selective Laser Melting (SLM): Microstructure, high cycle fatigue, and fracture behavior. Mater. Des..

[18] Zhang H., Zhu H., Qi T., Hu Z., Zeng X. (2016). Selective laser melting of high strength Al–Cu–Mg alloys: Processing, microstructure and mechanical properties. Mater. Sci. Eng. A.

[19] Martin J.H., Yahata B.D., Hundley J.M., Mayer J.A., Schaedler T.A., Pollock T.M. (2017). 3D printing of high-strength aluminium alloys. Nature.

[20] Qi T., Zhu H., Zhang H., Yin J., Ke L., Zeng X. (2017). Selective laser melting of Al7050 powder: Melting mode transition and comparison of the characteristics between the keyhole and conduction mode. Mater. Des..

[21] Nie X., Chen Z., Qi Y., Zhang H., Zhang C., Xiao Z., Zhu H. (2020). Effect of defocusing distance on laser powder bed fusion of high strength Al–Cu–Mg–Mn alloy. Virtual Phys. Prototyp..

[22] Schmidtke K., Palm F., Hawkins A., Emmelmann C. (2011). Process and Mechanical Properties: Applicability of a Scandium modified Al-alloy for Laser Additive Manufacturing. Phys. Procedia.

[23] Spierings A.B., Dawson K., Heeling T., Uggowitzer P.J., Schäublin R., Palm F., Wegener K. (2017). Microstructural features of Sc- and Zr-modified Al-Mg alloys processed by selective laser melting. Mater. Des..

[24] Spierings A.B., Dawson K., Kern K., Palm F., Wegener K. (2017). SLM-processed Sc- and Zr- modified Al-Mg alloy: Mechanical properties and microstructural effects of heat treatment. Mater. Sci. Eng. A.

[25] Cabrera-Correa L., González-Rovira L., de Dios López-Castro J., Botana F.J. (2022). Pitting and intergranular corrosion of Scalmalloy^®^ aluminium alloy additively manufactured by Selective Laser Melting (SLM). Corros. Sci..

[26] Cabrera-Correa L., González-Rovira L., de Dios López-Castro J., Castillo-Rodríguez M., Botana F.J.J.M.C. (2023). Effect of the heat treatment on the mechanical properties and microstructure of Scalmalloy^®^ manufactured by Selective Laser Melting (SLM) under certified conditions. Mater. Charact..

[27] Awd M., Tenkamp J., Hirtler M., Siddique S., Bambach M., Walther F. (2017). Comparison of Microstructure and Mechanical Properties of Scalmalloy^®^ Produced by Selective Laser Melting and Laser Metal Deposition. Materials.

[28] Wang Y., Liu H., Ma X., Wu R., Sun J., Hou L., Zhang J., Li X., Zhang M. (2019). Effects of Sc and Zr on microstructure and properties of 1420 aluminum alloy. Mater. Charact..

[29] Samuel A.M., Alkahtani S.A., Doty H.W., Samuel F.H. (2015). Role of Zr and Sc addition in controlling the microstructure and tensile properties of aluminum–copper based alloys. Mater. Des..

[30] Muhammad A., Xu C., Xuejiao W., Hanada S., Yamagata H., Hao L., Chaoli M. (2014). High strength aluminum cast alloy: A Sc modification of a standard Al–Si–Mg cast alloy. Mater. Sci. Eng. A.

[31] Taendl J., Orthacker A., Amenitsch H., Kothleitner G., Poletti C. (2016). Influence of the degree of scandium supersaturation on the precipitation kinetics of rapidly solidified Al-Mg-Sc-Zr alloys. Acta Mater..

[32] Nie X., Zhang H., Zhu H., Hu Z., Ke L., Zeng X. (2018). Effect of Zr content on formability, microstructure and mechanical properties of selective laser melted Zr modified Al-4.24Cu-1.97Mg-0.56Mn alloys. J. Alloys Compd..

[33] Zhang H., Zhu H., Nie X., Yin J., Hu Z., Zeng X. (2017). Effect of Zirconium addition on crack, microstructure and mechanical behavior of selective laser melted Al-Cu-Mg alloy. Scr. Mater..

[34] Lu Y., Zhang H., Xue P., Wu L., Liu F., Jia L., Ni D., Xiao B., Ma Z. (2023). Microstructural Evaluation and Tensile Properties of Al-Mg-Sc-Zr Alloys Prepared by LPBF. Crystals.

[35] Pan W., Zhai Z., Liu Y., Liang B., Liang Z., Zhang Y. (2022). Research on Microstructure and Cracking Behavior of Al-6.2Zn-2Mg-xSc-xZr Alloy Fabricated by Selective Laser Melting. Crystals.

[36] Leirmo J.L. (2021). High Strength Aluminium Alloys in Laser-Based Powder Bed Fusion—A Review. Procedia CIRP.

[37] Kotadia H.R., Gibbons G., Das A., Howes P.D. (2021). A review of Laser Powder Bed Fusion Additive Manufacturing of aluminium alloys: Microstructure and properties. Addit. Manuf..

[38] Liu D., Yürekli B., Ullsperger T., Matthäus G., Schade L., Nolte S., Rettenmayr M. (2021). Microstructural aspects of additive manufacturing of Al Li alloys with high Li content. Mater. Des..

[39] Li L., Meng X., Huang S., Wang H., Li P., Zhou J. (2022). Investigating the effect of the scanning speed on the characteristics of Al-Li alloy fabricated by selective laser melting. J. Manuf. Process..

[40] Xiao R., Zhang X. (2014). Problems and issues in laser beam welding of aluminum–lithium alloys. J. Manuf. Process..

[41] Qi Y., Zhang H., Nie X., Hu Z., Zhu H., Zeng X. (2020). A high strength Al–Li alloy produced by laser powder bed fusion: Densification, microstructure, and mechanical properties. Addit. Manuf..

[42] Qi Y., Hu Z., Zhang H., Nie X., Zhang C., Zhu H. (2021). High strength Al–Li alloy development for laser powder bed fusion. Addit. Manuf..

[43] Sun Z., He B., Li K., Tu Y., Wang H. (2022). Study on microstructure evolution and aging precipitation behavior of a novel Al-Li alloy fabricated by laser rapid melting. J. Alloys Compd..

[44] Yürekli B., Schade L., Ullsperger T., Seyfarth B., Kohl H., Matthäus G., Liu D., Rettenmayr M., Nolte S. (2020). Additive manufacturing of binary Al-Li alloys. Procedia CIRP.

[45] Wang A., Yan Y., Chen Z., Qi H., Yin Y., Wu X., Jia Q. (2022). Characterisation of the multiple effects of Sc/Zr elements in selective laser melted Al alloy. Mater. Charact..

[46] Mochugovskiy A.G., Mikhaylovskaya A.V. (2020). Comparison of precipitation kinetics and mechanical properties in Zr and Sc-bearing aluminum-based alloys. Mater. Lett..

[47] Robson J.D., Prangnell P.B. (2001). Dispersoid precipitation and process modelling in zirconium containing commercial aluminium alloys. Acta Mater..

[48] Mochugovskiy A.G., Tabachkova N.Y., Ghayoumabadi M.E., Cheverikin V.V., Mikhaylovskaya A.V. (2021). Joint effect of quasicrystalline icosahedral and L12-strucutred phases precipitation on the grain structure and mechanical properties of aluminum-based alloys. J. Mater. Sci. Technol..

[49] Ning J.L., Jiang D.M. (2007). Influence of Zr addition on the microstructure evolution and thermal stability of Al–Mg–Mn alloy processed by ECAP at elevated temperature. Mater. Sci. Eng. A.

[50] Jones M.J., Humphreys F.J. (2003). Interaction of recrystallization and precipitation: The effect of Al3Sc on the recrystallization behaviour of deformed aluminium. Acta Mater..

[51] Monastyrska T.O., Berezina A.L., Molebny O.A., Kotko A.V. (2021). Effect of alloying with transition metals on the aging of anomalously supersaturated solid solution of Al–Mg alloys. Appl. Nanosci..

[52] Zhai Z., Pan W., Liang B., Liu Y., Zhang Y. (2022). Cracking Behavior, Microstructure and Properties of Selective Laser Melted Al-Mn-Mg-Sc-Zr Alloy. Crystals.

[53] Yin P., Liu Y., Liang Z., Pan W., Shao S., Zhang Y. (2023). Microstructure, Mechanical Properties and Fracture Behavior of Micron-Sized TiB(2)/AlZnMgCu(Sc,Zr) Composites Fabricated by Selective Laser Melting. Materials.

[54] (2020). Methods for Chemical Analysis of Aluminium and Aluminium Alloys-Part 25: Determination of Elements Content—Inductively Coupled Plasma Atomic Emission Spectrometric Method.

[55] Qu M., Guo Q., Escano L.I., Nabaa A., Hojjatzadeh S.M.H., Young Z.A., Chen L. (2022). Controlling process instability for defect lean metal additive manufacturing. Nat. Commun..

[56] (2010). Metallic Materials-Tensile Testing—Part 1 : Method of Test at Room Temperature.

[57] Wang P., Deng L., Prashanth K.G., Pauly S., Eckert J., Scudino S. (2018). Microstructure and mechanical properties of Al-Cu alloys fabricated by selective laser melting of powder mixtures. J. Alloys Compd..

[58] Wang Z., Lin X., Kang N., Hu Y., Chen J., Huang W. (2020). Strength-ductility synergy of selective laser melted Al-Mg-Sc-Zr alloy with a heterogeneous grain structure. Addit. Manuf..

[59] Wang Z., Lin X., Kang N., Wang Y., Yu X., Tan H., Yang H., Huang W. (2021). Making selective-laser-melted high-strength Al–Mg–Sc–Zr alloy tough via ultrafine and heterogeneous microstructure. Scr. Mater..

[60] Spierings A.B., Dawson K., Voegtlin M., Palm F., Uggowitzer P.J. (2016). Microstructure and mechanical properties of as-processed scandium-modified aluminium using selective laser melting. CIRP Ann..

[61] Kurz W., Bezençon C., Gäumann M. (2001). Columnar to equiaxed transition in solidification processing. Sci. Technol. Adv. Mater..

[62] Gäumann M., Henry S., Cléton F., Wagnière J.D., Kurz W. (1999). Epitaxial laser metal forming: Analysis of microstructure formation. Mater. Sci. Eng. A.

[63] Jia Z.-h., RØYset J., Solberg J.K., Liu Q. (2012). Formation of precipitates and recrystallization resistance in Al–Sc–Zr alloys. Trans. Nonferrous Met. Soc. China.

[64] Kim J.-H., Jeun J.-H., Chun H.-J., Lee Y.R., Yoo J.-T., Yoon J.-H., Lee H.-S. (2016). Effect of precipitates on mechanical properties of AA2195. J. Alloys Compd..

